# Rietveld Refinement of Electron Diffraction Patterns of Nanocrystalline Materials Using MAUD: Two-Beam Dynamical Correction Implementation and Applications

**DOI:** 10.3390/ma18030650

**Published:** 2025-02-01

**Authors:** Ankur Sinha, Valentino Abram, Luca Lutterotti, Stefano Gialanella

**Affiliations:** 1 Department of Industrial Engineering, University of Trento, Via Sommarive 9, 38123 Trento, Italy; luca.lutterotti@unitn.it (L.L.); stefano.gialanella@unitn.it (S.G.); 2 Department of Mathematics, University of Trento, Via Sommarive 14, 38123 Trento, Italy; valentino.abram@unitn.it

**Keywords:** nanocrystalline materials, electron diffraction, Rietveld refinement

## Abstract

Nanocrystalline (NC) materials have widespread industrial usage. X-ray and neutron diffraction techniques are primary tools for studying the structural and microstructural features of NC materials. Selected area electron diffraction (SAED) patterns collected using a transmission electron microscope (TEM) on polycrystalline nanostructured materials, featuring nested rings, that are analogous to Debye–Scherrer patterns, possess similar potentials to aid materials characterisation. The utility of SAED patterns is further enhanced by the possibility of applying crystallographic approaches, like full pattern fitting procedures, based on Rietveld refinement algorithms, enabling the evaluation of material features, such as crystallite size, lattice distortions, defect structures, and the presence of secondary phases even from very small volume scale. In this paper, we have discussed the possibilities afforded by a Rietveld code applied to SAED patterns of NC materials, including the mathematical implementation of the two-beam dynamical correction model in MAUD software (version 2.9995), and a critical discussion of the results obtained on different NC materials.

## 1. Introduction

Since the seminal work by Gleiter and co-authors [1], nanostructured materials have gained continued prominence, largely due to their tuneable properties, reported to be superior to some extent or different from those of the micro grain-sized (>1 μm) counterparts. For instance, bulk NC and thin films exhibit high values of hardness [2,3], strength [4,5], and fatigue resistance [6,7].

Quantitative characterisation of materials is a prerequisite for any application. The analysis of diffraction line profile is a versatile and non-destructive approach to study the microstructure of a NC material. In this regard, a reliable quantification is possible considering the whole diffraction pattern based on the Rietveld algorithm [8] to obtain the best fit between the measured and calculated diffraction profiles. Although the bulk of the research using the Rietveld refinement characterisation route belongs to X-ray diffraction and neutron diffraction [9,10], still, the applicability, advantages, and constraints of Rietveld refinement of SAED patterns to characterise NC materials have been discussed in our previous papers [11,12,13,14] and also by other researchers [15]. The advancements in the literature prioritising structural/microstructural evaluation of nanostructured polycrystalline materials based on Rietveld refinement of SAED patterns must be attributed to the development of dedicated software. The initial studies on this topic were done under “kinematical approximation” [16] for NC materials, in which dynamical scattering was substantially disregarded, based to the small interaction volumes.

Weirich et al. [17] studied the structure of nanocrystalline anatase using first the ELD program [18] to obtain averaged radial intensity curves from representative sectors of the powder patterns, and subsequent Rietveld refinement was performed using FULLPROF [19] with integrated intensities. Similar techniques were adopted by Weirich et al. [20] in their second paper on the structure determination of NC anatase, for NC MnFe_2_O_4_ by Kim et al. [21], and for hydroxyapatite nano-powder by Song et al. [22]. Tonejc et al. [23] studied the evolution of crystallite size and lattice parameters dependence in nanocrystalline anatase using the “Process Diffraction” software (version 1.2) developed by Lábár [24] for deducing circularly integrated electron diffraction (ED) intensities, in association with FULLPROF for the Rietveld refinement. Oleynikov et al. [25] developed the program TexPat, which is suitable to handle textured electron diffraction patterns for unit–cell determination and structure solution. Gemmi et al. [26] used the software “Material Analysis Using Diffraction” (MAUD) developed by Lutterotti [27,28] for texture analysis of ring electron diffraction patterns. The authors demonstrated that built-in electron scattering factors in MAUD and those imported into MAUD using JEMS software [29] under the kinematic approximation of electron diffraction produced identical results. In all the previously listed works, no dynamical correction [30] was implemented, still providing satisfactory results for the characterisation of truly nanocrystalline samples. In this perspective, electron structure factors were used for the evaluation of the diffracted intensities.

Li [31] developed a dedicated program: “PCED” for the simulation of polycrystalline electron diffraction patterns and phase identification. In one of the latest versions of the software [32], Blackman two-beam dynamical correction was also included. Lábár published three papers [15,33,34], based on his software “Process Diffraction”, and the final version included Blackman correction for dynamical effects too. As far as MAUD is concerned, Boullay et al. [35] demonstrated that two-beam dynamical correction implemented in the software yielded very accurate results, and the approach was also extended to textured NC specimens. For a binary system, Serafini et al. [11] showed the higher sensitivity of the Rietveld refinement of SAED over XRD in the detection and quantification of a minority crystalline phase at the nanoscale. Further, Sinha et al. published a two-part paper focusing on the correct instrumental broadening function determination of the electron microscope [12] and on elucidating the microstructural features of different nanocrystalline materials through electron diffraction [13]. The approach was extended even to complex particulate matter emissions collected on a dynamometer test rig from tribological experiments on materials for brake linings [14].

The advantages of analysing the SAED patterns under two-beam dynamical correction have been discussed by different research groups [13,26,32,35]. However, the implementation of the relevant correction in the software to account for the multiple scattering has not yet been discussed, even in the case of MAUD [35]. The mathematics to arrive to the final formula, although complicated, is essential for a better understanding of the results obtained at the end of the refinement cycle [36]. This paper concerns studying the microstructure/structure of NC materials through Rietveld refinement of SAED patterns with the implemented Blackman correction model. We have also demonstrated the consequences of the implementation of the correction model on the overall Rietveld fitting and the significant specimen parameters that govern kinematical and dynamical scattering. The NC materials studied are CeO_2_, Si, CoFe_2_O_4_, Au-Ag, and NaYF_4_.

## 2. Dynamical Scattering Approximation as Implemented in MAUD

We report here a brief description of the model implemented in MAUD to calculate the single layer approximation for the dynamical electron scattering calculation of the structure factor. The single layer approximation was originally developed by Blackman [30] and further clarified by other authors (Self et al. [37], Peng [38], Sinkler et al. [39], and Li [40]), but still, not all details are reported in a comprehensive way for a rigorous demonstration and for the implementation in computer code. We successfully derived the approximation from a solution of the Schrödinger’s equation, but the length of it made it necessary to delegate the details to a separate paper [36]. We are giving here only the necessary formulas for the computer code implementation. The meanings of different symbols used in these formulae are given in Table 1.

The solution of the Schrödinger’s equation using the kinematical theory gives us that the intensity of a reflection for a sample of thickness *T* is equal to

(1) $$I_{T}^{kin}=\frac{8m^{2}V_{q}^{2}}{V_{c}^{2}\hbar^{4}{\left\|k\right\|}^{2}}T$$

If we define the extinction distance as

(2) $$\xi_{q}\overset{\cdot }{=}\frac{\hbar^{2}V_{c}\left\|k\right\|}{2meV_{q}}$$

we obtain

(3) $$I_{T}^{kin}=\frac{2T}{\xi_{q}^{2}}$$

For the scattering off a lattice using the dynamical theory, the solution of the Schrödinger’s equation using Bloch waves and, as argued in [30], considering that the excitation error ξ is varied (as in the kinematical case), we finally obtain

(4) $$I_{T}^{dyn}=\frac{2}{\xi_{q}}\int_{-\frac{\pi }{4}}^{\frac{\pi }{4}}d\theta_{2}\int_{0}^{A\left(\theta_{2}\right)}J_{0}\left(2v\right)\;dv$$

where

(5) $$A=A\left(\theta_{2}\right)=\frac{\pi T}{cos\left(\theta_{2}\right)\xi_{q}}$$

and $J_{0}$ denotes the zeroth-order Bessel function of the first kind. Notice then that, if we consider the ratio between $I_{T}^{dyn}$ and $I_{T}^{kin}$, we have

(6) $$\frac{I_{T}^{dyn}}{I_{T}^{kin}}=\frac{\xi_{q}}{T}\int_{-\frac{\pi }{4}}^{\frac{\pi }{4}}d\theta_{2}\int_{0}^{A\left(\theta_{2}\right)}J_{0}\left(2v\right)\;dv$$

In the MAUD software code, we first calculate the structure factor for the kinematical approximation $F_{hkl}^{kin}$ using electron scattering factors from Peng et al. [41]. We recall here (Equation (1) of [38]) the relation between the Fourier coefficient $V_{q}$ and the electronic atomic scattering factor $F_{q}$:

(7) $$F_{q}=\frac{2me}{\hbar^{2}}V_{q}$$

From it and defining the crystal thickness *T* as a refinable parameter, Equation (6) can be used to calculate the structure factor for the dynamical approximation as

(8) $${F_{hkl}^{dyn}}^{2}={F_{hkl}^{kin}}^{2}\int_{-\frac{\pi }{4}}^{\frac{\pi }{4}}d\theta_{2}\int_{0}^{A\left(\theta_{2}\right)}J_{0}\left(2v\right)\;dv$$

where $A\left(\theta_{2}\right)$ is becoming

(9) $$A\left(\theta_{2}\right)=\frac{F_{hkl}^{kin}}{V_{c}cos\left(\theta_{2}\right)}\frac{meT\pi }{2\hbar^{2}K\cdot 10^{20}}$$

The factor $10^{20}$ is necessary if Å is used for both $V_{c}$ and *T*. Finally, K is calculated as

(10) $$K=\sqrt{\frac{2m}{\hbar^{2}}\left(E_{k}+\frac{eF_{000}^{kin}}{V_{c}}\right)}$$

## 3. Materials and Methods

TEM data were collected with a STEM-EDXS Talos F200S (Thermo Fisher Scientific, Waltham, MA, USA) field emission source instrument, operated at 200 kV. For the acquisition of the SAED patterns, the specimen was first set at the eucentric height of the stage, and the objective lens was set to its standard focus. Then, a representative region of the specimen was selected, avoiding the possibility of contributions from the TEM grid. Further, the astigmatism of the condenser and the objective lenses was duly compensated, the final focusing condition of the BF image was confirmed with the help of fast Fourier transform, and diffraction astigmatism, if encountered during the acquisition of SAED pattern, was also corrected. It is important to minimise the deviation of the camera length (CL) from the calibrated values [12] to determine reliable microstructural/structural parameters.

The SAED patterns were acquired using selected area (SA) apertures of sizes 40, 200, or 800 μm with instrumental camera lengths (CL) of 658, 844, 1080, or 1360 mm. The instrumental broadening function of different SA aperture–CL combinations has been determined in a previous work [12]. The SAED patterns were indexed using Process Diffraction open-source software (version 8.7.1) [42]. The MAUD program [43] was used for the Rietveld refinement of the SAED patterns. The readers are advised to download the latest MAUD version from https://maud.radiographema.com accessed on 10 August 2024.

The methodology of performing Rietveld refinement on 2D SAED patterns has been described in our previous papers [12,13]. In brief, the intensity integration along the Debye rings of the 2D pattern is performed using the ImageJ (version 2.x, https://imagej.net/ij/ accessed on 15 October 2017) plugin implemented in MAUD. This allows the direct import of the SAED pattern in MAUD, without the need for external processing, and preserves each data point’s original coordinate position. The portions of the SAED pattern masked by the beam stopper can be removed manually. Multiple diffractograms are generated by azimuthal segmentation of the rings into equal sectors, which are used to refine the displacement in the image centre and the tilting errors. The 1D plot of experimental intensities shown in the Rietveld analyses is built by integrating all the diffractograms.

A close monitoring of the weighed sum of the squared differences (WSS) and weighed profile R-factor without background (${Rwp}_{no\_bkg}$) [12], to obtain their minimum value with a minimum set of refined parameters, along with the visual inspection of the Rietveld fitting [44], were the criteria to inspect the analysis at successive refinement stages.

The sample preparation route for NC CeO_2_, Si, NaYF_4_, and CoFe_2_O_4_ was relatively straightforward. A small amount of each one of the nano-powders was dispersed in ethanol using ultrasonic mixing for 10 min. Thereafter, a few drops of the suspension were deposited onto a carbon-coated Cu (300 mesh) TEM grid, and the suspension was left to dry in air. The NC Au-Ag sample was prepared as described in Reference [45].

## 4. Results and Discussion: Rietveld Refinement of SAED Patterns

### 4.1. NC CeO_2_ and Si

**Figure 1:**
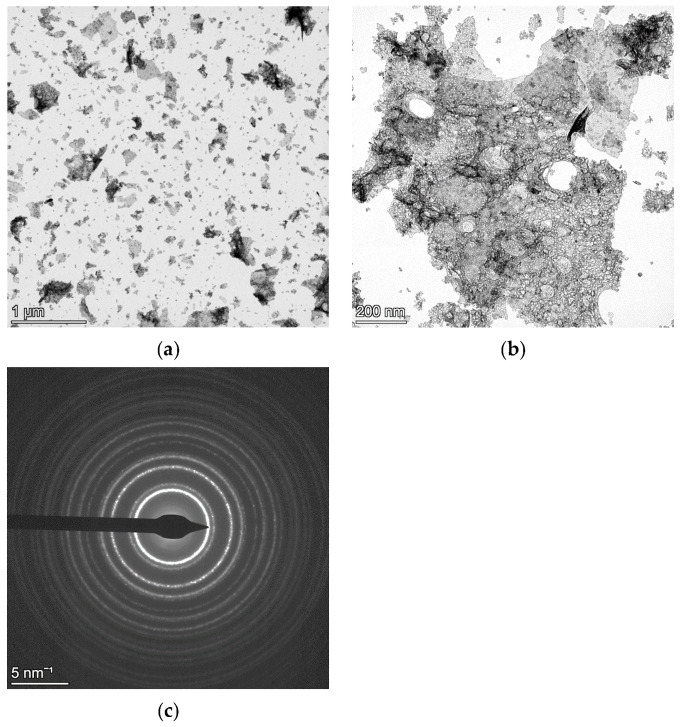
Low magnification BF image of NC CeO_2_ showing small agglomerates of particles (**a**). A relatively higher magnification BF image (**b**) with the corresponding SAED pattern from the field of view in (**c**).

**Figure 2:**
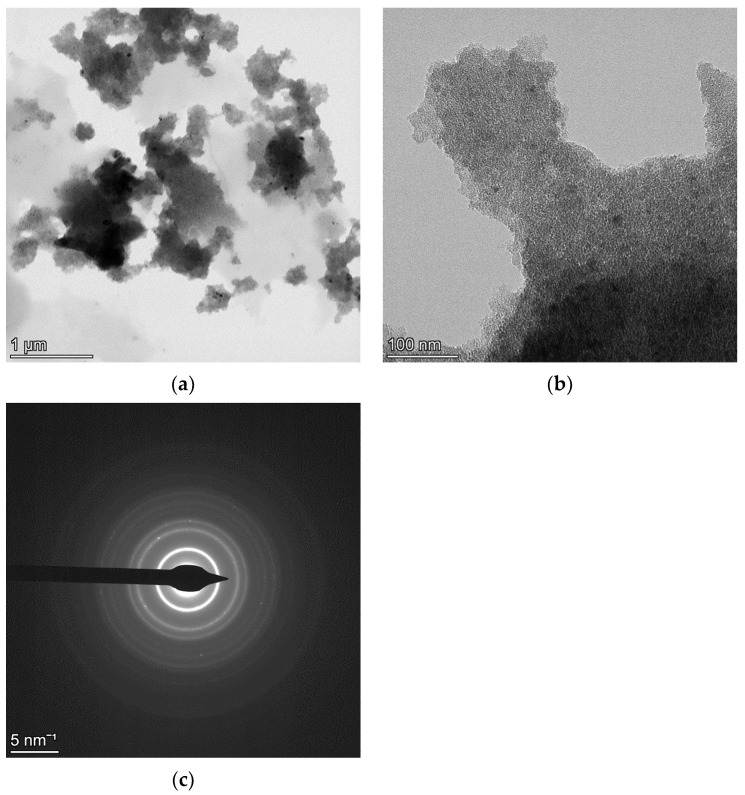
Low magnification BF image of NC Si showing large agglomerates of particles (**a**). A relatively higher magnification BF image (**b**) with the corresponding SAED pattern from the field of view in (**c**).

Figure 1 and Figure 2 refer to TEM data of NC CeO_2_ and NC Si, respectively. The low magnification bright-field (BF) images in Figure 1a for CeO_2_ and in Figure 2a for Si have been collected to show the presence of very large agglomerates in the Si specimen, as compared to those of CeO_2_. The SAED pattern in Figure 1c for CeO_2_ and in Figure 2c for Si have been collected from the field of view of their respective relatively high magnification BF images in Figure 1b and Figure 2b.

**Figure 3:**
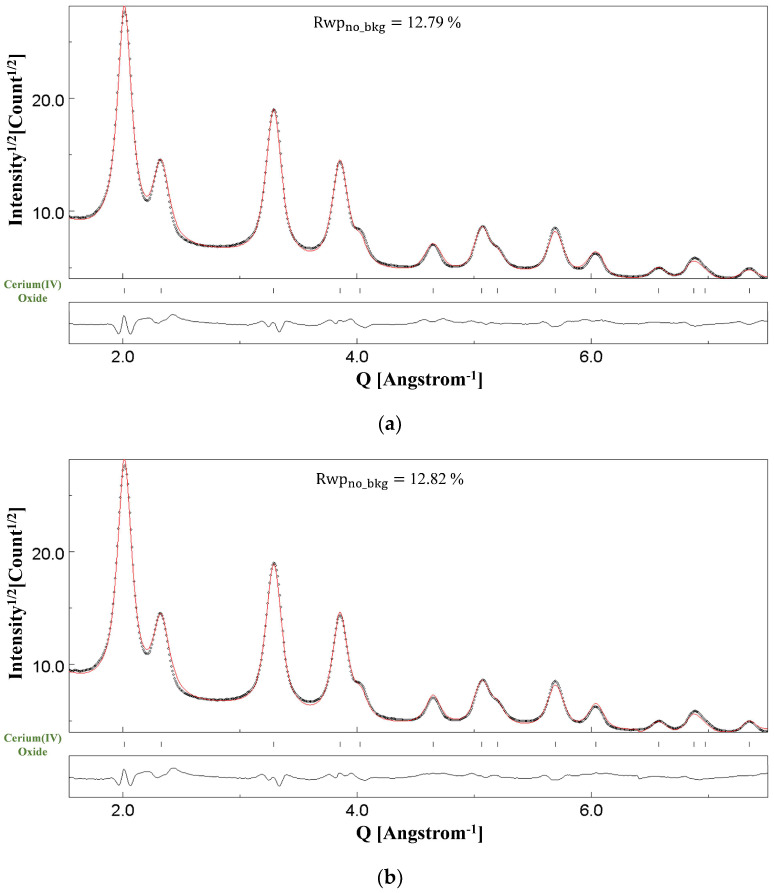
Results of the Rietveld refinements of the NC-CeO_2_ sample under (**a**) the two-beam dynamical correction (${Rwp}_{no\_bkg}=$ 12.79%) and (**b**) the kinematical approximation (${Rwp}_{no\_bkg}=$ 12.82%). The dotted line is the experimental intensity profile, and the red continuous line is the calculated profile.

**Figure 4:**
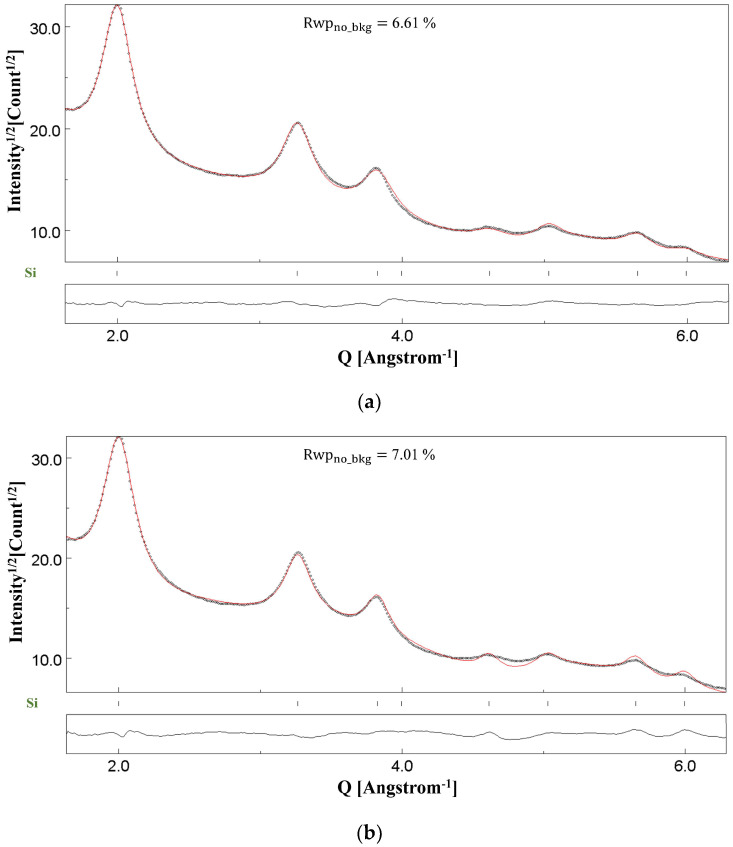
Results of Rietveld refinements of the NC-Si sample under (**a**) the two-beam dynamical correction (${Rwp}_{no\_bkg}=$ 6.61%) and (**b**) the kinematical approximation (${Rwp}_{no\_bkg}=$ 7.01%). The dotted line is the experimental intensity profile, and the red line continuous is the calculated profile.

Figure 3 shows the Rietveld fitting of the SAED pattern of NC CeO_2_ in Figure 1c. The Rietveld fitting shown in Figure 3a was obtained upon implementation of the two-beam dynamical correction, whereas Figure 3b, referring to the same SAED as Figure 3a, shows the refinement under the kinematical approximation. Figure 4 shows the Rietveld fitting of the SAED pattern of NC Si shown in Figure 2c. For the NC Si sample, there is a clear difference in the fittings at the end of the refinement cycles for two-beam correction (Figure 4a) and kinematical approximation (Figure 4b). In the Rietveld plots shown, the black dots represent the experimental intensities determined based on the conversion of the two-dimensional SAED diffraction patterns to one-dimensional plot of the scattered intensity as a function of the scattering vector, *Q* $\left(=\frac{4\pi }{\lambda }\sin\theta \right)$, where 2θ is the position of the Bragg reflections and *λ* is the wavelength of the incident electrons. The short vertical bars alongside different phases in each figure represent the positions of the Bragg reflections. The bottom curve (continuous black line) represents the difference plot between the experimentally observed pattern and the calculated pattern.

We have used NC CeO_2_ for the determination of the instrumental broadening function of our instrument in a previous work [12], wherein detailed microstructural evaluation has also been done using XRD. However, in this section, the point of inquiry is the difference in the Rietveld fittings under the two fitting models for Si and CeO_2_ samples. The consequences of the results are even more interesting, because the average crystallite size of NC CeO_2_ is of the order of 120 Å [12], whereas NC Si has a relatively smaller size of 30 Å [13], and it has been generally proposed in the literature that, for small-sized nanoparticles and thin films, a microstructural evaluation even under the kinematical approximation leads to reliable results [21,46].

Table 2 for NC CeO_2_ and Table 3 for NC Si list the calculated values of the kinematical ($F_{KIN})$ and dynamical ${(F}_{DYN})$ structure factors for different (*hkl*) planes. For both samples, the ratio of the two structure factors has also been determined. The practically constant value for ${Ratio}_{F}$ (maximum deviation = 0.03) in the case of NC CeO_2_ in Table 2, determined as the ratio of $F_{DYN}$ to $F_{KIN}$, proves the prevailing role of quasi-kinematical scattering [20]. On the other hand, as seen in Table 3 for NC Si, the ratio of the amplitudes of the structure factors varies between a maximum value of 0.64 to a minimum of 0.48. This variation is an indication of the occurrence of dynamical scattering.

In MAUD, dynamical correction is implemented by refining the thickness parameter *T* discussed in Section 2. The refined value of this thickness parameter of course cannot exactly match the physical thickness of the TEM specimen that will vary from one region to another for NC samples but is indeed related. For NC CeO_2_, a smaller value of *T* as 45(1) Å was obtained, whereas, for NC Si, featuring coarser agglomerates, a much higher value of 5008(42) Å was determined at the final refinement scale. In principle, the refined thickness parameter *T* should be close to the actual sample thickness value. However, the model that we currently use in MAUD is an approximation (two-beam dynamical correction). As a consequence, a deviation from a real *T* can occur to compensate for model deficiencies. A better approximation like the multi-beam model would be better in describing the multiple scattering but is too computing-intensive to be employed in Rietveld refinements at the moment. A high degree of coupling between dynamical scattering and the refined Debye–Waller factor for electron diffraction experiments [46] also contributes to the deviation from the real value of the thickness parameter.

In view of the above-mentioned results, it seems that the critical parameter supporting the kinematical scattering is a smaller thickness of the specimen. For the NC Si sample, the presence of thick agglomerates leads to multiple-electron scattering, and hence, two beam dynamical diffraction correction needs to be taken into account for a good fitting of the SAED pattern for reliable microstructural evaluation. On the other hand, for the NC CeO_2_ sample, although agglomerates are present, their size thickness is relatively smaller. A key finding of our previous work [12] was that the Rietveld fittings of CeO_2_ SAED patterns collected using different SA apertures—800 μm, 200 μm, and 40 μm and, analysed under kinematical approximation and two-beam dynamical correction, yielded identical results. Although a relatively very large number of crystallites would contribute to the diffraction pattern collected from the field of view in Figure 1a, the thickness and the size of the agglomerates are small, and hence, the effects of dynamical scattering are substantially attenuated. However, as seen in Figure 2a,b for the NC Si sample, the thickness and size of agglomerates are relatively larger, and hence, dynamical scattering is bound to occur, thereby making the inclusion of two beam correction a mandatory step for a reliable fitting and relevant microstructural evaluation.

Another significant point worth mentioning in view of the observed results for the two samples is an anisotropic crystallite size. Although both the samples display some anisotropy [12,13], still the dominant factor causing dynamical scattering is the thickness of the agglomerates.

The effect of dynamical scattering on the Debye–Waller factor [47], deserves to be discussed further. It has been proposed in the literature that, although the Debye–Waller factor can be refined using electron powder diffraction data, it should be handled with caution due to its interaction with other fitting parameters, especially the dynamical scattering component [15,46]. We attempted to refine a common value of the Debye–Waller factor for both Ce and O during the refinement cycle of NC CeO_2_ and obtained a value of 0.027(7). Indeed, as compared to those obtained for XRD data, the refined value of the Debye–Waller factor has a higher magnitude for electron diffraction data collected on NC materials. For the NC CeO_2_ sample, we obtained a value of 0.0024(3) for the fitting of the XRD pattern [12], which is a decrement of roughly 10 times. Similarly, for a NC Y_2_O_3_ sample reported elsewhere [13], XRD data and SAED data refinements yielded values of 0.010(5) and 0.02(2) respectively. The reasons for higher Debye–Waller factors for electron diffraction is the local heating, as demonstrated by Reimer [48], a high surface-to-volume ratio of NC materials, defects generated because of rearrangement of atoms caused due to “knock-on damage” by the incoming electron beam [46]. For the NC Si sample, an even higher value of 0.04(5) for the Debye–Waller factor was determined.

### 4.2. NC CoFe_2_O_4_

**Figure 5:**
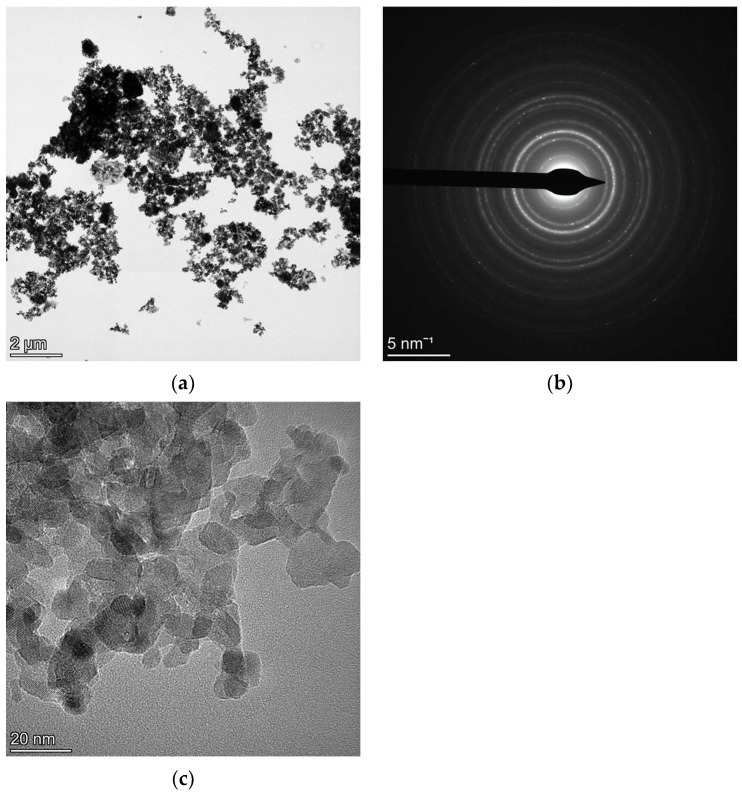
BF image showing the presence of thick NC CoFe_2_O_4_ agglomerates (**a**) with the SAED pattern from the field of view in (**b**). A high magnification image with overlapping grains (**c**).

Figure 5a shows a BF image of NC CoFe_2_O_4_, in which thick agglomerates of particles can be clearly seen. The spotty ring SAED pattern in Figure 5b was collected from the field of view of the BF image. Figure 5c is a high-magnification image showing overlapping grains with significant size variations.

**Figure 6:**
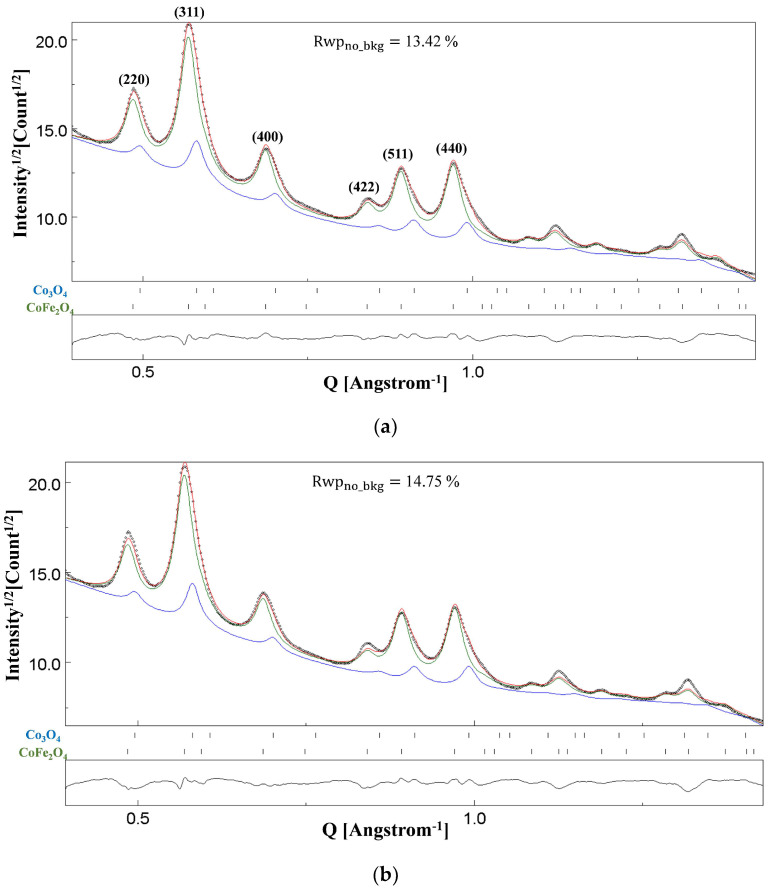
Results of Rietveld refinements of the NC CoFe_2_O_4_ sample under (**a**) the two-beam dynamical correction (${Rwp}_{no\_bkg}=$ 13.42%) and (**b**) the kinematical approximation (${Rwp}_{no\_bkg}=$ 14.75%). The dotted line is the experimental intensity profile, and the red line is the calculated profile. Blue and green lines correspond to Co_3_O_4_ and CoFe_2_O_4_ phases, respectively.

Figure 6a,b show the Rietveld fittings of the SAED pattern in Figure 5b under two-beam dynamical correction and kinematical approximation, respectively. The blue and green lines in these figures represent the calculated profiles for Co_3_O_4_ and CoFe_2_O_4_ phases, respectively. The red line is the combined calculated pattern, which is plotted against the experimental pattern (black dots). It may not be clear immediately from the visual inspection that the profile fitting in Figure 6a is better, although some indications are visible, for instance, the indexed (220) reflection having a better match between the experimental and calculated intensities. However, a lower reliability factor value, ${Rwp}_{no\_bkg}$ = 13.42%, as compared to a higher value of 14.75% for the fitting in Figure 6b justifies that the SAED pattern collected from such a specimen with thick agglomerates must be analysed with the implemented correction. The refined value of the thickness parameter *T* (Equation (1) in Section 2) was obtained as 1588(24) Å.

The microstructure of this sample is interesting, since the Rietveld refinement of the SAED pattern supports the presence of two phases. Some of the CoFe_2_O_4_ reflections have been indexed in Figure 6a. By visual inspection, we can infer that the broadening and shapes of the peaks, more evident for the (400) and (440) reflections, may not be solely due to a single phase. We do not know the synthesis route adopted for this commercial CoFe_2_O_4_ nano-powder; however, a possible method is the solid-state technique, with the precursors being cobalt (Co_3_O_4_) and iron (Fe_2_O_3_) oxides. The end product is not CoFe_2_O_4_ only. Owing to insufficient ball milling time and/or subsequent calcination time/temperature, a remnant fraction of the precursor oxide may be found in the final product [49]. To investigate this aspect, as concerns in particular some unconverted Co_3_O_4_, we performed the analyses in the Rietveld fittings shown in Figure 6a,b with dual phases, i.e., CoFe_2_O_4_ and Co_3_O_4_, and indeed the result shows that broadening of the peaks may imply the existence of both phases, for which the Crystallographic Information File (CIF) cards n. 5910063 for CoFe_2_O_4_ and n. 9005895 for Co_3_O_4_ were used.

We were further interested in determining the occupancies of Fe and Co at different atomic sites in the two phases, as they would have changed continuously with Co_3_O_4_ being converted to CoFe_2_O_4_. To keep the number of the parameters to be refined optimal so that the results could be logically interpreted, even to avoid overprocessing the diffraction data, we adopted the following methodology. For CoFe_2_O_4_, at the atomic site of Co, Fe was added, and vice versa was done for the Fe site. Only the occupancy of Fe at one of the sites was refined, and Fe occupancy at the other site was set equal to this refined value. The summation of occupancies at both the sites was set to one, i.e., Fe + Co occupancy = 1. For Co_3_O_4_, at the two atomic sites of Co, we also added Fe and employed the same refinement strategy as done for CoFe_2_O_4_. For each of the two phases, the lattice parameter was then determined as a function of its Fe occupancy:

a (Å)=8.1975+X(8.39−8.1975)

where 8.1975 Å is the unrefined lattice parameter of Co_3_O_4_, 8.39 Å is the unrefined lattice parameter of CoFe_2_O_4_, and X being the refined Fe occupancy in the respective phases. These values, along with other structural/microstructural parameters and phase fractions, have been listed in Table 4 and Table 5 for the fitting with the two-beam dynamical correction and kinematical approximation models, respectively. For CoFe_2_O_4_, using the dynamical correction model, we get a refined value of the oxygen position closer to the literature value (see Table 4). In particular, the mean value of the x (atomic position) parameter from the literature is around 0.38. The two-beams approximation gives a final refined value quite close, while the deviation for the kinematical approximation is unusually large for a Rietveld refinement. In addition, Rietveld fitting with the dynamical two-beam correction model yields a lower reliability factor.

Using the two-beam dynamical model, we do not expect higher precision in the parameter values, since that depends more on the measurements, noise, etc. However, we should instead improve the accuracy thanks to the less approximated model. The cell parameters are not very accurate by electron diffraction compared to X-ray or neutron, so these have been bound to the atomic occupancies as explained earlier. Consequently, the precision of the lattice parameters determination is bound to the precision (estimated standard deviations as determined from the algorithm) of the atomic occupancies in our analysis.

In reality, the trick was implemented to achieve exactly the contrary, to improve the accuracy of atomic occupancies bounding them to the cell parameters. Even if these last are not as precise as in X-ray or neutron diffraction, they are much more sensitive than atomic occupancies as we remind the reader that electron/X-ray diffraction only feels electron densities in the lattice and not specific atomic species. One electron scattering difference, as we observe for the couple Co/Fe, is lost in the diffraction intensities as an example. For the same reason, we typically do not refine H positions.

For both the Rietveld fittings shown in Figure 6, the isotropic size–strain model was used, since the inclusion of the Popa model [50] for anisotropic size–strain did not significantly improve the results. Even with the isotropic size–strain model, there was no apparent improvement in the fittings upon refining the r.m.s. microstrain parameter, and hence, it was set to zero. The average crystallite sizes of ~77 Å and ~82 Å were determined for CoFe_2_O_4_ and Co_3_O_4,_ respectively. A phase fraction of ~20% for Co_3_O_4_ suggests that a substantial amount of this phase could not be converted into the desired NC CoFe_2_O_4_. Hence, based on the results of the Rietveld refinement, we can conclude that the synthesis route adopted for the manufacture of this commercial CoFe_2_O_4_ nano-powder yielded a two-phase composition. For each phase, the microstructural/structural parameters are distinguishable.

### 4.3. Au-Ag

**Figure 7:**
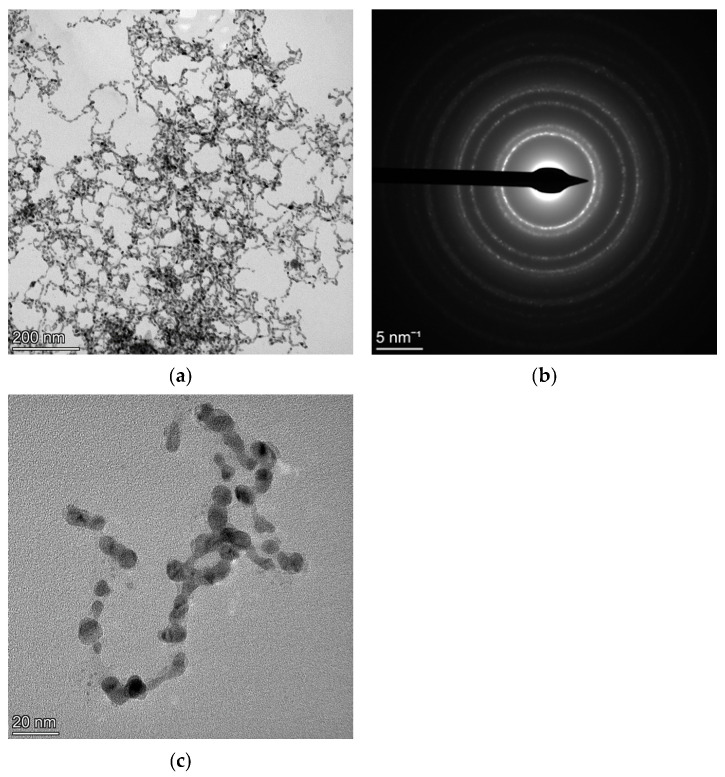
BF image showing the presence of thin Au-Ag agglomerates (**a**) with the SAED pattern from the field of view in (**b**). A high-magnification image showing the orientation of selected overlapping grains (**c**).

An Au-Ag nanoalloy sample was synthesised by Pini et al. [45] for solar energy harvesting applications. In this section, we are interested in understanding the microstructure of the elongated particles seen in the BF image (Figure 7a), referred to as “nanocorals” by the authors, using the SAED pattern shown in Figure 7b. Figure 7c is a high magnification image in which selected overlapping grains can be seen.

**Figure 8:**
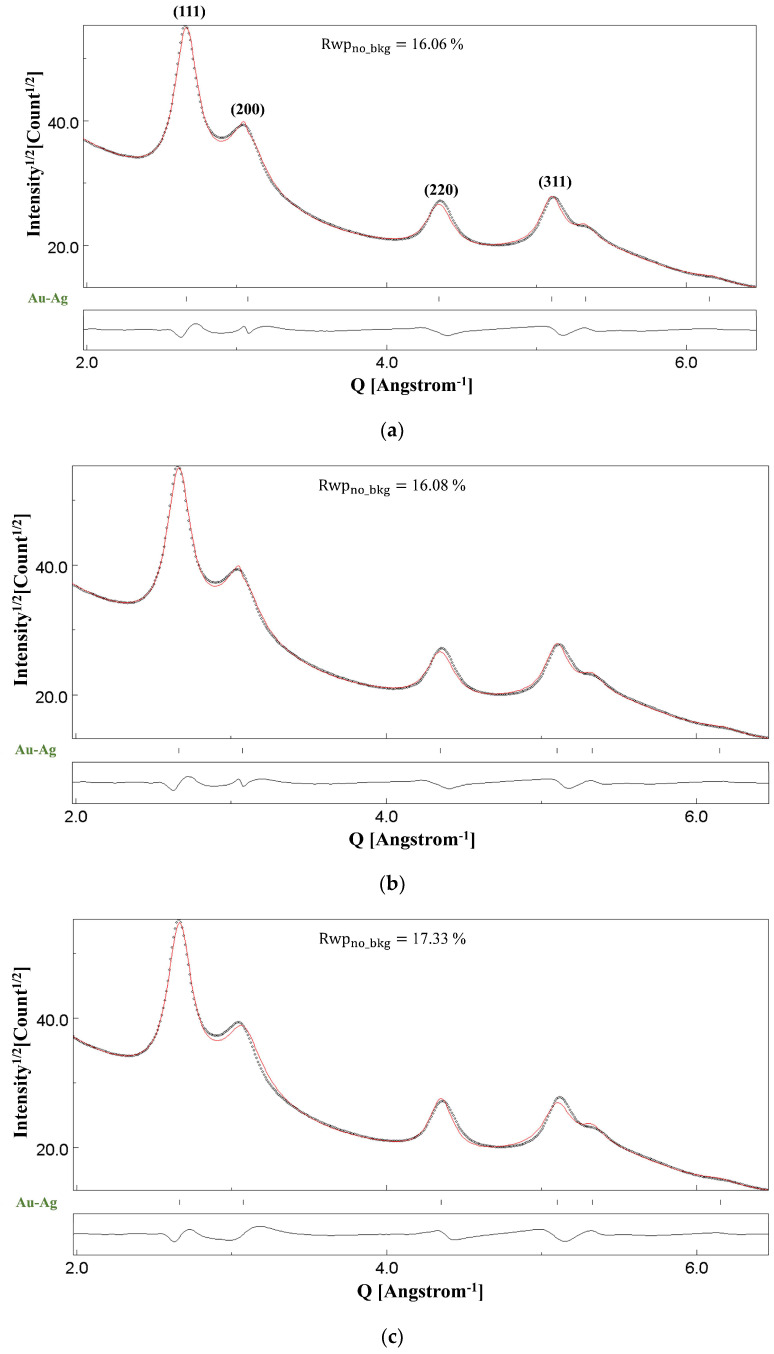
Results of Rietveld refinements of the Au-Ag sample under (**a**) the two-beam dynamical correction (${Rwp}_{no\_bkg}=$ 16.06%), (**b**) the kinematical approximation (${Rwp}_{no\_bkg}=$ 16.08%), and (**c**) the two-beam dynamical correction but without accounting for planar defects (${Rwp}_{no\_bkg}=$ 17.33%). The dotted line is the experimental intensity profile, and the red continuous line is the calculated profile.

Figure 8a shows the Rietveld fitting of the SAED pattern in Figure 7b, having very broad peaks. Gold and silver have identical crystal structures—both crystallise in the fcc structure—have comparable atomic sizes and lattice parameters, and form a solid solution over the entire range of composition [51,52]. Hence, it is not possible to distinguish between an Au-Ag nanoalloy from either of the two monometallic Au and Ag phases using diffraction line broadening. This means that the position of the indexed (111), (200), (220), and (311) reflections in Figure 8a might correspond either to Au, Ag, or Au-Ag alloy. However, the formation of the substitutional solid solution has already been established in [45], and hence, we determined the lattice parameter of this nanoalloy as a function of Au composition. The technique adopted for this sample was similar to that for NC CoFe_2_O_4_, and the lattice parameter of the nanoalloy was determined as a (Å)=4.07825+X(4.0862−4.07825), where 4.07825 Å is the unrefined lattice parameter of Au, 4.0862 Å is the unrefined lattice parameter of Ag, and X is the refined Au occupancy in the nanoalloy. These values, along with the anisotropic crystallite sizes, are given in Table 6 for the Rietveld refinement shown in Figure 8a, with the Blackman correction implemented. The refined values of the r.m.s. microstrain parameter were negligible and hence set to zero. The CIF files used for the Rietveld refinement correspond to card numbers 9008463 and 9008459 of the Crystallographic Open Database (COD) [53] for Au and Ag, respectively. The atomic occupancy of Au in the nanoalloy determined at the final stage of the refinement routine was 0.91(7).

If we compare the relatively thinner agglomerates of the Au-Ag sample as seen in Figure 7a,c with those of CoFe_2_O_4_ or Si illustrated in previous sections, we expect a smaller value of the thickness parameter *T*. Indeed, a smaller value of 83(26) (Å) was obtained at the final refinement stage shown in Figure 8a. Consequently, the Rietveld refinement shown in Figure 8b carried out under only kinematical approximation yields identical fitting. Thus, through this Au-Ag sample too, we can conclude that, in specimens with smaller thicknesses and agglomerates, dynamical diffraction is substantially attenuated. The results from the Rietveld fittings with kinematical approximation, similar to those with the Blackman correction implemented, are listed in Table 7.

The microstructure of the Au-Ag sample is interesting for the presence of planar defects. Planar defects can cause broadening and shifts of the diffraction peaks, as discussed originally by Warren [54]. Figure 8c shows the Rietveld fitting obtained with the Blackman correction implemented but without refining the planar defect parameters, namely intrinsic deformation fault probability ${(\alpha }^{\prime })$, extrinsic deformation fault probability ${(\alpha }^{\prime \prime })$, and twin fault probability ${(\beta }^{\prime })$ [55]. There is clear evidence of line shifts and broadening in the profile caused by the presence of the stacking and twin faults. Thus, the overall broadening of the peaks is a result of both smaller crystallite sizes and the presence of planar defects. The refined values of ${(\alpha }^{\prime }),\;{(\alpha }^{\prime \prime }),\;and\;{(\beta }^{\prime })$ have been listed in Table 8 for the profile in Figure 8a. The magnitude of these faulting probabilities are of the order reported for experiments involving ball milling [56]. This indicates that the synthesis route of Au-Ag nanocorals adopted by Pini et al. is generating planar defects in the synthesised nanoalloy. Their presence is confirmed by high-resolution TEM images (see Figure 3 in [45]).

Line defects, like dislocations, influence r.m.s. microstrains, which can be determined as the standard deviation of the interplanar spacing from its mean value. However, the refined value of the microstrain was negligible, also with the presence of stacking faults. This result aligns with the Warren theory, since the effect of stacking faults is similar to that of small coherent domains, having no elastic stress field around them, except at their boundaries [57].

Rietveld refinement of X-ray powder diffraction pattern has been used previously in many studies to assess the presence of stacking faults. However, a similar methodology can be extended to SAED patterns collected from localised sample regions, as demonstrated in this section.

### 4.4. NC NaYF_4_

**Figure 9:**
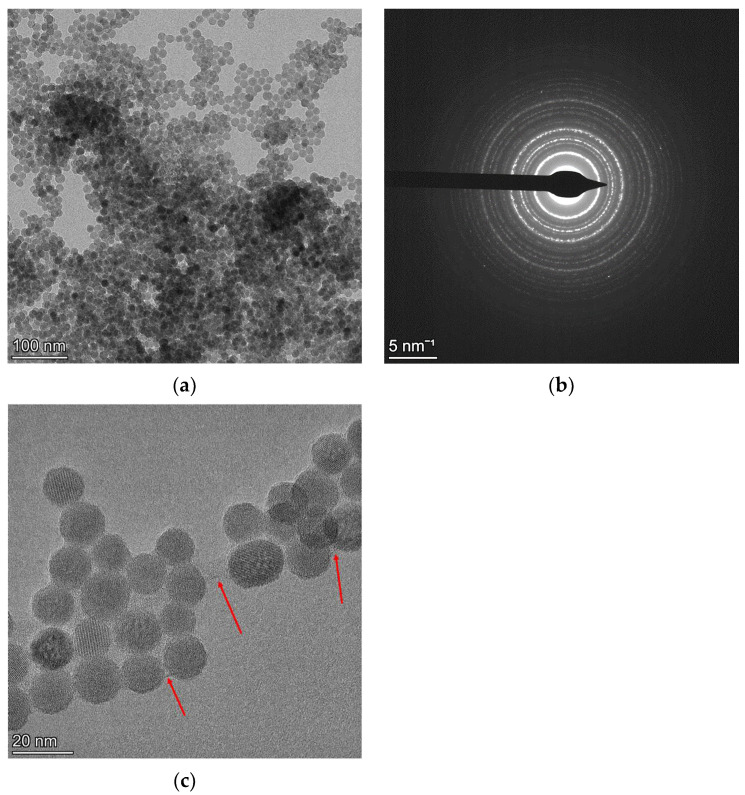
BF image showing the presence of thin NC NaYF_4_ agglomerates (**a**) with the SAED pattern from the field of view in (**b**). A high magnification image showing the orientation of some individual grains with the presence of a film shown using red arrows (**c**).

Figure 9a is a BF image of the NaYF_4_ nano-powder, and from its field of view, the SAED pattern shown in Figure 9b was acquired. The grains of this nano-powder display largely spherical morphology. Although any one grain could comprise several crystallites, the high magnification image in Figure 9c was analysed using ImageJ software, and an average “crystallite size” of 135 Å was determined. In some areas, a thin film is observed between the grains, arrowed in the figure. The presence of this film also influences the analysis of the SAED pattern, as it will be highlighted below.

**Figure 10:**
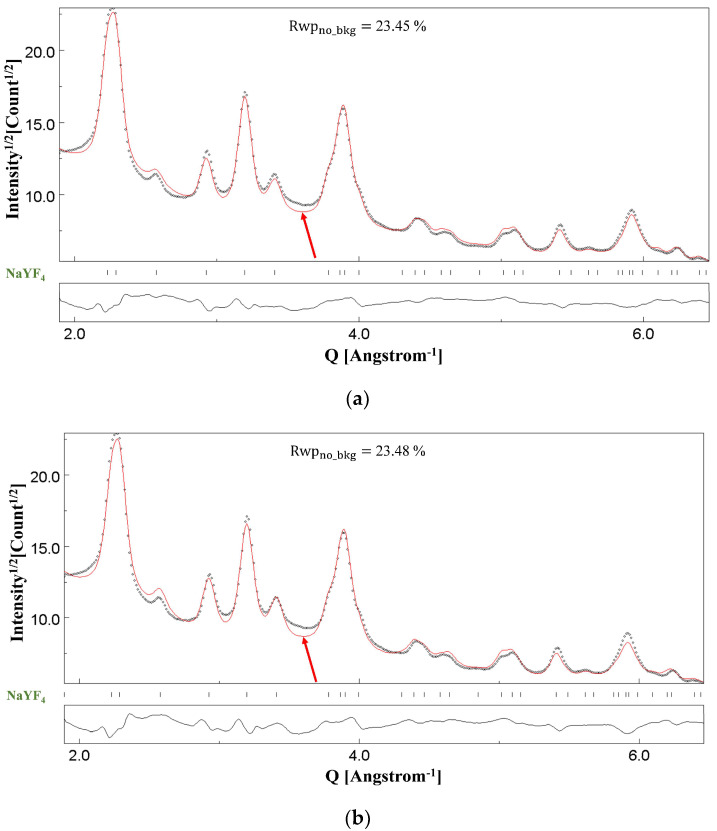
Results of Rietveld refinements of the NC NaYF_4_ sample under (**a**) the two-beam dynamical correction (${Rwp}_{no\_bkg}=$ 23.45%) and (**b**) the kinematical approximation (${Rwp}_{no\_bkg}=$ 23.48%). The dotted line is the experimental intensity profile, and the red line is the calculated profile. The red arrows in the figure show the effect of the amorphous thin film (see Figure 9c) on the Rietveld fitting.

NaYF_4_ crystalline cell symmetry can be either hexagonal or cubic. The NaYF_4_ sample studied in this research has a hexagonal symmetry, and CIF file number 1517673 downloaded from the COD [53] was used in the Rietveld refinements [58]. Figure 10a,b show the Rietveld refinement of the SAED pattern in Figure 9b under two-beam dynamical correction and kinematical approximation, respectively. The fittings look identical upon visual inspection, and the same can also be inferred from the reliability factors: ${Rwp}_{no\_bkg}=23.45\%$ in Figure 10a and ${Rwp}_{no\_bkg}=23.48\%$ in Figure 10b. Microstructural/structural parameters obtained at the final refinement cycle with the Blackman correction implemented and with kinematical approximation have been listed in Table 9 and Table 10, respectively. The refined values of the atomic positions are very similar for the two fitting models.

A reliable fitting could be obtained only with the assumption of isotropic crystallite size, without the need to introduce the Popa model [50] for anisotropic crystallites. The crystallite sizes evaluated from Rietveld refinements as 140 Å (kinematical approximation) and 142 Å (two-beam correction) match closely with the evaluated value of 135 Å from the high magnification image in Figure 9c. Also, in this case, the refined values of the r.m.s. microstrain parameter were negligible and set to zero. Moreover, again, a relatively smaller value of the thickness parameter *T* as 139(44) (Å) was determined.

The effect of the amorphous carbonaceous thin film seen in Figure 9c is like that of the supporting film on the TEM grids, discussed already in our previous paper [12]. For NC NaYF_4_, the diffused contribution from the thin film regions seen in Figure 9c has not been accounted for, and hence, its effect on the Rietveld fitting can be seen (see arrows in Figure 10).

Finally, Table 11 summarises the refined values of the thickness parameter for all studied NC materials for quick reference.

## 5. Conclusions and Perspectives

In this paper, we demonstrated the applicability of Rietveld refinement to the microstructural characterisation of nanocrystalline materials using SAED ring patterns. Physical chemical properties of a NC material are directly related to its microstructure and hence, morphology, crystallite size, defects, and presence of secondary phases can have substantial effects on the performance. In this perspective, the presence of cobalt oxide as a secondary phase in CoFe_2_O_4_ nano-powder, and the existence of planar defects in the Au-Ag sample, are significant factors when assessing materials properties.

The thickness and size of the agglomerates of NC specimens from which the SAED patterns are acquired are the dominant factors that determine whether a reliable quantification is possible just with kinematical approximation or if the two-beam dynamical correction is required. Specimens with a relatively smaller value of the refined thickness parameter *T*-CeO_2_: 45(1) Å, Ag-Au: 83(26) Å, and NaYF_4_: 139(44) Å displayed identical Rietveld fittings under kinematical approximation and Blackman correction models. However, NC materials having specimens with a relatively higher value of refined thickness parameters *T*-CoFe_2_O_4_: 1588(24) Å and Si: 5008(42) Å clearly showed a different quality of the fittings under the two refinement models. This was confirmed through visual inspection of the whole pattern and by the reliability factors determined at the final refinement stage. In conclusion, specimens with thick and larger agglomerates should be studied with the Blackman formulation implemented for reliable quantitative characterisation of a microstructure/structure.

Although, at the nanoscale, anisotropy in terms of crystallite size and defect concentrations is substantially reduced [59,60], for some NC materials, we had to adopt an anisotropic crystallite size model, resulting in a better and more reliable Rietveld fitting. Still, anisotropic crystallite size is not a dominant factor favouring dynamical scattering. This was clear from the Rietveld fittings of the Au-Ag sample, with irregularly shaped crystallites seen from TEM micrographs (Figure 7a,c) and anisotropic crystallite sizes determined from the diffraction line broadening (Table 6), still yielding identical results from the two fitting models. For the CoFe_2_O_4_ sample, dynamical scattering is enhanced, since the material specimen has thick and large agglomerates, although a reliable fitting was obtained considering only the isotropic size–strain model (Table 4).

Our study can also serve to provide an indirect assessment of the thickness of a TEM specimen, which is always a critical issue, using the refined thickness parameter *T*. Such an approach could be extended to bulk (electron transparent) NC specimens, for which the thickness values are known beforehand, and a comparative study could be performed.

## Figures and Tables

**Table 1:** Symbols used in different equations and their meanings.

Symbol	Meaning
$V_{c}$	cell volume
m	electron mass
e	electron charge
$\left\|k\right\|=\frac{2\pi }{\lambda }$	wave vector of the incident electron
E $,\;E_{k}=\frac{\hbar^{2}}{2m}{\left\|k\right\|}^{2}$	energy of the electron
$V_{q}$	q-th Fourier coefficient of the potential *V*
T	thickness of the sample or crystal
$\xi_{q}=\frac{\hbar^{2}V_{c}\left\|k\right\|}{2meV_{q}}$	q-th extinction distance

**Table 2:** Comparison of dynamical structure factor amplitudes (*F_DYN_*), kinematical structure factor (*F_KIN_*), and their ratios (*Ratio_F_*) after Rietveld refinement for different reflections of NC CeO_2_.

Plane	*F_DYN_* (Å)	*F_KIN_* (Å)	*Ratio_F_*
111	160.06	152.64	1.05
200	92.37	89.79	1.03
220	180.61	171.32	1.05
311	164.43	156.67	1.05
222	59.29	58.22	1.02
400	83.45	81.36	1.03
331	110.25	106.66	1.03
420	68.16	66.76	1.02

**Table 3:** Comparison of dynamical structure factor amplitudes (*F_DYN_*), kinematical structure factor amplitudes (*F_KIN_*), and their ratios (*Ratio_F_*) after Rietveld refinement for different reflections of NC Si.

Plane	*F_DYN_* (Å)	*F_KIN_* (Å)	*Ratio_F_*
111	34.44	69.38	0.50
220	33.27	54.97	0.61
311	28.38	44.39	0.64
400	15.79	25.18	0.63
331	18.36	32.57	0.56
422	19.79	41.27	0.48

**Table 4:** Cell parameters, crystallite sizes, atomic occupancies, atomic position (O), phase fractions, thickness parameter *T*, and reliability factors as evaluated from the Rietveld refinement of NC CoFe_2_O_4_ with the Blackman correction implemented. Fe and Co atomic occupancies in both CoFe_2_O_4_ and Co_3_O_4_ phases correspond to X and (1 − X), respectively.

Phase	Lattice Parameter a (Å)	Crystallite Size (Å)	Atomic Occupancy	Atomic Position (Oxygen)	Phase Fraction (%)	Thickness (Å)	Rwp_no_bkg_ (%)
CoFe_2_O_4_	8.383	76.9(6)	Co: 0.032 Fe: 0.967(5)	0.3815(4)	79.74	1588(24)	13.42
Co_3_O_4_	8.20	82(2)	Co: 0.97 Fe: 0.03(40)	-	20.26		

**Table 5:** Cell parameters, crystallite sizes, atomic occupancies, atomic position (O), phase fractions, and reliability factors as evaluated from the Rietveld refinement of NC CoFe_2_O_4_ with the kinematical approximation. The Fe and Co atomic occupancies in both the CoFe_2_O_4_ and Co_3_O_4_ phases correspond to X and (1 − X), respectively.

Phase	Lattice Parameter a (Å)	Crystallite Size (Å)	Atomic Occupancy	Atomic Position (Oxygen)	Phase Fraction (%)	Rwp_no_bkg_ (%)
CoFe_2_O_4_	8.380	75.9(2)	Co: 0.048 Fe: 0.951(5)	0.3896(2)	82.75	14.75
Co_3_O_4_	8.20	96(1)	Co: 0.98 Fe: 0.02(40)	-	17.25	

**Table 6:** Cell parameters, atomic occupancy (Au), crystallite sizes, thickness parameter *T*, and reliability factor as evaluated from the Rietveld refinement of Au-Ag sample with the Blackman correction implemented.

Lattice Parameter (Å) and Atomic Occupancy of Au	Crystallite Size, Average (Å)	Anisotropic Crystallite Size (Å)	Thickness (Å)	Rwp_no_bkg_ (%)
4.08; 0.91(7)	127(6)	[200]: 35 [420]: 81	83(26)	16.06

**Table 7:** Cell parameters, atomic occupancy (Au), crystallite sizes, and reliability factor as evaluated from the Rietveld refinement of Au-Ag sample with the kinematical approximation.

Lattice Parameter (Å) and Atomic Occupancy of Au	Crystallite Size, Average (Å)	Anisotropic Crystallite Size (Å)	Rwp_no_bkg_ (%)
4.08; 0.90(1)	126(2)	[200]: 35 [420]: 81	16.08

**Table 8:** Probabilities of stacking faults in the Au-Ag sample as determined from the Rietveld fitting in Figure 8a.

Intrinsic Deformation Fault Probability (*α*′)	Extrinsic Deformation Fault Probability (*α*″)	Twin Fault Probability (*β*′)
0.050(2)	0.001(1)	0.002(6)

**Table 9:** Cell parameters, atomic positions, crystallite size, thickness parameter *T*, and reliability factor as evaluated from the Rietveld refinement of the NC NaYF_4_ sample with the Blackman correction implemented.

Lattice Parameter (Å)	Refined Atomic Positions	Crystallite Size, Average (Å)	Thickness (Å)	Rwp_no_bkg_ (%)
	Na (2)—z: 0.438(7)	142(1)	139(44)	23.45
a: 5.6298(6)	F (1)—x: 0.666(1); y: 0.086(2)			
c: 3.3221(6)	F (2)—x: 0.712(1); y: 0.756(2)			

**Table 10:** Cell parameters, atomic positions, crystallite size, and reliability factor as evaluated from the Rietveld refinement of the NC NaYF_4_ sample with the kinematical approximation.

Lattice Parameter (Å)	Refined Atomic Positions	Crystallite Size, Average (Å)	Rwp_no_bkg_ (%)
	Na (2)—z: 0.439(7)	140(1)	23.48
a: 5.6296(6)	F (1)—x: 0.665(1); y: 0.084(2)		
c: 3.3220(5)	F (2)—x: 0.714(1); y: 0.759(2)		

**Table 11:** Refined thickness parameter *T* for different NC materials.

Material	Thickness Parameter, *T* (Å)
CeO_2_	45(1)
Si	5008(42)
CoFe_2_O_4_	1588(24)
Au-Ag	83(26)
NaYF_4_	139(44)

## Data Availability

The original contributions presented in this study are included in the article. Further inquiries can be directed to the corresponding author.
